# 2016 Annual Report of the University of Kansas Health System Poison Control Center

**Published:** 2018-05-18

**Authors:** Stephen L. Thornton, Lisa Oller, Doyle M. Coons

**Affiliations:** University of Kansas Health System Poison Control Center, Kansas City, KS

**Keywords:** drug overdose, poisoning, ingestion, toxicology

## Abstract

**Introduction:**

This is the 2016 Annual Report of the University of Kansas Health System Poison Control Center (PCC). The PCC is one of 55 certified poison control centers in the United States and serves the state of Kansas 24-hours a day, 365 days a year, with certified specialists in poison information and medical toxicologists. The PCC receives calls from the public, law enforcement, health care professionals, and public health agencies. All calls to the PCC are recorded electronically in the Toxicall® data management system and uploaded in near real-time to the National Poison Data System (NPDS), which is the data repository for all poison control centers in the United States.

**Methods:**

All encounters reported to the PCC from January 1, 2016 to December 31, 2016 were analyzed. Data recorded for each exposure includes caller location, age, weight, gender, substance exposed to, nature of exposure, route of exposure, interventions, medical outcome, disposition and location of care. Encounters were classified further as human exposure, animal exposure, confirmed non-exposure, or information call (no exposure reported).

**Results:**

The PCC logged 21,965 total encounters in 2016, including 20,713 human exposure cases. The PCC received calls from every county in Kansas. The majority of human exposure cases (50.4%, n = 10,174) were female. Approximately 67% (n = 13,903) of human exposures involved a child (defined as age 19 years or less). Most encounters occurred at a residence (94.0%, n = 19,476) and most calls (72.3%, n = 14,964) originated from a residence. The majority of human exposures (n = 18,233) were acute cases (exposures occurring over eight hours or less). Ingestion was the most common route of exposure documented (86.3%, n = 17,882). The most common reported substance in pediatric encounters was cosmetics/personal care products (n = 1,362), followed by household cleaning product (n = 1,301). For adult encounters, sedatives/hypnotics/antipsychotics (n = 1,130) and analgesics (n = 1,103) were the most frequently involved substances. Unintentional exposures were the most common reason for exposures (81.3%, n = 16,836). Most encounters (71.1%, n = 14,732) were managed in a non-healthcare facility (i.e., a residence). Among human exposures, 14,679 involved exposures to pharmaceutical agents while 10,176 involved exposure to non-pharmaceuticals. Medical outcomes were 32% (n = 6,582) no effect, 19% (n = 3,911) minor effect, 8% (n = 1,623) moderate effect, and 2% (n = 348) major effects. There were 15 deaths in 2016 reported to the PCC. Number of exposures, calls from healthcare facilities, cases with moderate or major medical outcomes, and deaths all increased in 2016 compared to 2015.

**Conclusion:**

The results of the 2016 University of Kansas Health System Poison Control annual report demonstrates that the center receives calls from the entire state of Kansas totaling over 20,000 human exposures per year. While pediatric exposures remain the most common, there is an increasing number of calls from healthcare facilities and for cases with serious outcomes. The experience of the PCC is similar to national data. This report supports the continued value of the PCC to both public and acute health care in the state of Kansas.

## Introduction

This is the 2016 Annual Report of University of Kansas Health System Poison Control Center (PCC). The PCC is a 24-hour 365 day/year health care information resource serving the state of Kansas. It was founded in 1982 and is certified with the American Association of Poison Control Centers (AAPCC). Currently, there are 55 certified poison control centers in the United States. The PCC is staffed by 10 certified specialists in poison information who are either critical care trained nurses or doctors of pharmacy. There is 24-hour back up provided by board certified medical toxicologists. The PCC receives calls from the public, law enforcement, health care professionals, and public health agencies. Encounters may involve an exposed animal or human (Exposure Call) or a request for information with no known exposure (Information Call). The PCC follows all cases to make management recommendations, monitor case progress, and document medical outcome. This information is recorded electronically in the Toxicall® data management system and uploaded in near real-time to the National Poison Data System (NPDS). NPDS is the data warehouse for all of the nation’s poison control centers.^1^ The NPDS utilizes a products database that contains over 427,000 products to classify exposures. The database is maintained and updated continuously by data analysts at the Micromedex Poisindex® System.^1^ The average time to upload data for all PCs is 9.52 minutes, creating a real-time national exposure database and surveillance system.^1^ The PCC has the ability to share NPDS real time surveillance with state and local health departments and other regulatory agencies. What follows is analysis and summary of all encounters reported to the PCC from January 1, 2016 to December 31, 2016.

## Methods

All PCC encounters recorded electronically in the Toxicall® data management system from January 1, 2016 to December 31, 2016 were analyzed. Cases were first classified as either an exposure or suspected exposure (Human Exposure, Animal Exposure, Non-Exposure Confirmed Cases) or a request for information with no reported exposure (Information Call). Data extracted includes caller location, age, weight, gender, exposure substance, number of follow-up calls, and nature of exposure (i.e., unintentional, recreational, or intentional). Additional data collected included exposure scenario, route of exposure (oral, dermal, parenteral), interventions, medical outcome (no effect, minor, moderate, severe, or death), disposition (admitted to noncritical care unit, admitted to critical care unit, admitted to psychiatry unit, lost to follow-up, or treated and released) and location of care (non-health care facility or health care facility). For this analysis, a pediatric case was defined as any patient 19 years of age or less. This is consistent with NPDS methodology. For medical outcome, the following definitions were used: minor - minimally bothersome symptoms, moderate - more pronounced symptoms, usually requiring treatment, and major life threatening signs and symptoms.

Data were analyzed using Microsoft Excel (Microsoft Corp, Redmond, WA).

## Results

The PCC logged 21,965 total calls in 2016, including 20,713 human exposure cases, 87 non-exposure confirmed cases, 112 animal exposure cases, and 1,053 information calls. For information calls, drug information (n = 308) was most common reason for calling. Table 1 further describes the encounter types. The PCC made 32,137 follow-up calls in 2016. Follow-up calls were done in 60.9% of human exposure cases. One follow-up call was made in 29.5% of human exposure cases and multiple follow-up calls (range 2 – 44) were made in 31.3% of cases. In human exposure calls for which follow-up calls were made, an average of 2.54 follow-up calls per case were performed.

The PCC received calls from all 105 counties in Kansas. The county with the most number of calls was Sedgwick County with 3,358. In addition, calls were received from 47 states, the District of Columbia, and 12 calls were from foreign countries, including Turkey and Uganda.

**Figure 1:**
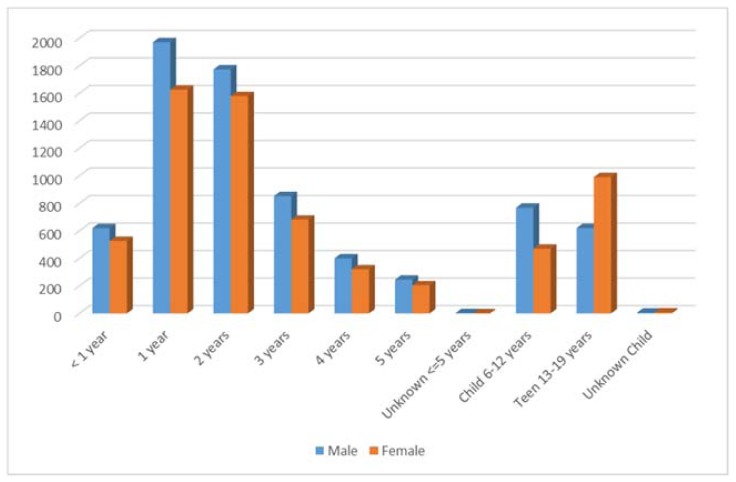
Distribution of human exposures by gender in children < 19 years old.

**Figure 2:**
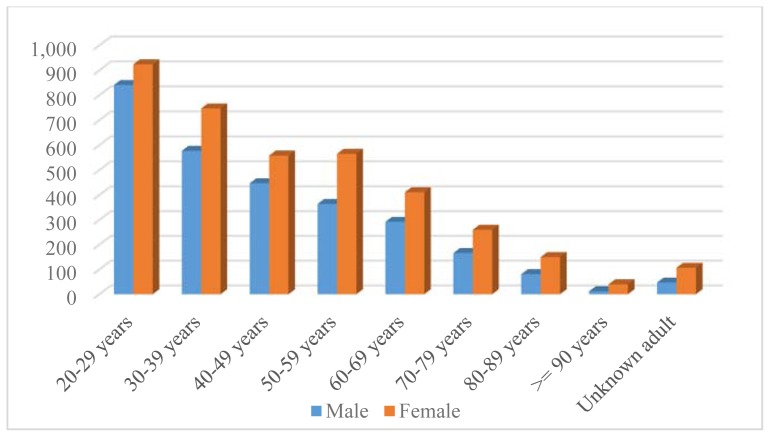
Distribution of human exposures by gender, adults > 20 years old.

The majority of human exposure cases (50.4%, n = 10,174) were female. A male predominance was found among encounters involving children younger than 13 years of age, but this gender distribution was reversed in teenagers and adults, with females comprising the majority of reported exposures. Approximately 67% (n = 13,903) of human exposures involved a child (defined as age 19 years or less). Table 2 illustrates distribution of human exposures by age and gender. Figure 1 demonstrates that patients 1 year of age were the most common age group involved in encounters reported to the PCC. For adults, the age group of 20 – 29 years old was encountered most commonly (Figure 2). Seventy-five (75) exposures occurred in pregnant women (0.4% of all human exposures). Of these exposures, 26.7% occurred in the first trimester, 42.7% occurred in the second trimester, and 28.0% occurred in the third trimester. Most of these exposures (78.7%) were unintentional exposures and 12.0% were intentional exposures. There were no reported deaths to PCC in pregnant women in 2016.

For human exposures, 72.3% (n = 14,964) of calls originated from a residence (own or other), while 94.0% (n = 19,476) of these exposures actually occurred at a residence (own or other). Calls from a health care facility accounted for 21.7% (n = 4,500) of human exposure encounters. Table 3 further details the origin of human exposure calls and where the exposure took place.

The majority of human exposures (n = 18,233) were acute cases (exposures occurring over eight hours or less). Chronic exposures (exposures occurring > 8 hours) accounted for 1.6% (327) of all human exposures reported. Acute on chronic exposures (single exposure that was preceded by a chronic exposure > 8 hours) totaled 2063 (9.96%). Ingestion was the most common route of exposure documented (86.3%, n = 17,882) in all cases (Table 4).

The most common reported substance in those less than 5 years of age was cosmetics/personal care products (n = 1,362) followed closely by household cleaning products (n = 1,301). For adult (> 20 years of age) encounters, sedatives/hypnotics/antipsychotics (n = 1,130) and analgesics (n = 1,103) were the most frequently involved substances. Among all encounters, analgesics (n = 2,813, 11%) were the most frequently encountered substance category. Table 5 lists most frequently encountered substance categories for pediatric encounters and Table 6 lists those for adult encounters. [A summary log for all exposures categorized by category and sub-category of substance is available with the manuscript on the website: kjm.kumc.edu].

There were a total of 399 plant exposures reported to the PCC. The most common plant exposure encountered was to pokeweed (*Phytolacca Americana*; n = 48). Table 7 lists the top 5 most encountered plants.

Unintentional exposures were the most common reason for exposures (81.3%, n = 16,836) while intentional exposures accounted for 16.3% (n = 3,377) of exposures. Table 8 lists reasons for human exposures. A majority of unintentional exposures (n = 10,897) occurred in the less than 5 years old age group. Up to age 12, 98.9% (n = 12,171) of ingestions were unintentional. However, in the 13 – 19 year-old group, intentional exposure was most common (63.1%, n = 1,087). In total, suspected suicide attempts accounted for 11.7% (n = 2,415) of human encounters. When a therapeutic error was the reason for exposure, a double dose was the most common scenario (n = 775).

Most encounters (71.1%, n = 14,732) were managed in a non-health care facility (i.e., a residence). Of the 5,747 encounters managed at a health care facility, 42% (n = 2419) were admitted. Table 9 lists the management site of all human encounters.

Among human exposures, 14,679 involved exposures to pharmaceutical agents, while 10,176 involved exposure to non-pharmaceuticals. Because an encounter could include both a pharmaceutical agent and non-pharmaceutical agent, this total is greater than the total number of encounters. However, 88.5% (n = 18,327) of all human exposures were exposed to only a single substance. Among these single substance exposures, the reason for exposure was intentional in 19.3% (n = 3,527) of pharmaceutical-only cases compared to 3.5% (n = 641) of non-pharmaceutical single substance exposures.

When medical outcomes were analyzed, 32% (n = 6,582) of human exposures had no effect, 19% (n = 3,911) had minor effect, 8% (n = 1,623) had moderate effect, and 2% (n = 348) major effects. Moderate and major effects were more common in those over 20 years of age and in those with intentional encounters. More serious outcomes were related to single-substance pharmaceutical exposures, accounting for 66.7% (n = 10) of the fatalities. Table 10 lists all medical outcomes by age and Table 11 lists them by reason for exposure.

Use of decontamination and specific therapies, including antidotal therapy, is detailed in Tables 12a and 12b.

There were 15 deaths in 2016 reported to the PCC. Fourteen of the deaths involved patients 20 years of age or older. Fourteen of the death cases involved intentional exposures. Table 13 details the 15 reported deaths.

Table 14 compares key statistics from 2015 to 2016. Number of exposures, calls from healthcare facilities, moderate or major outcomes and deaths increased from 2015.

## Discussion

The University of Kansas Health System Poison Control Center has been in operation for 35 years and serves the state of Kansas 24 hours a day, 365 days a year. Receiving over 26,000 calls per year, the PCC is an integral part of the emergency medical response, public health and health care facilities in Kansas. Childhood poisonings, both unintentional and intentional, are a major focus, with calls for patients under 19 years of age accounting for approximately 2/3 of all exposures.

The PCC statistics are similar to those seen nationally.^1^ In 2016, 2,710,042 encounters were logged by poison control centers nationwide, including 2,159,032 human exposures. Total encounters showed a 2.9% decline from 2015, but healthcare facility (HCF) human exposure cases increased by 3.6% from 2015. More serious outcomes (moderate, major or death) also increased. Nationwide, the five substance classes most frequently involved in adult exposures were analgesics, sedative/hypnotics/antipsychotics, antidepressants, cardiovascular drugs, and cleaning substances, while the top five most common exposures in children age 5 years or less were cosmetics/personal care products, household cleaning substances, analgesics, foreign bodies/toys/miscellaneous, and topical preparations. There were 1,415 exposure related fatalities reported nationwide in 2016.

The ongoing importance of the PCC is reflected in current trends that have seen rates of poisonings and overdoses increase at an alarming rate. The PCC saw an increase in number of calls from healthcare facilities, cases with moderate or major medical outcomes and deaths in 2016 compared to 2015. In an August 2017 report, the National Center for Health Statistic noted that the age-adjusted drug-poisoning death rate increased from 6.1 per 100,000 in 1999 to 16.3 per 100,000 in 2015, totaling over 50,000 deaths in 2015.^3^ Teenage (age 15 – 19) overdose deaths are increasing as well.^4^ The ongoing “opioid epidemic” is a major driver in the rise of poisoning deaths.^3^

Reporting exposures to the PCC is voluntary and the PCC is not contacted for all poisonings in the state of Kansas. Furthermore, in a majority of cases there is no objective confirmation of exposure. These limitations should be noted when interpreting PCC data.

## Conclusion

The results of the 2016 University of Kansas Health System Poison Control annual report demonstrated that the center receives calls from the entire state of Kansas, totaling over 20,000 human exposures per year. While pediatric exposures remain the most common, there is an increasing number of calls from healthcare facilities and for cases with serious outcomes. The experience of the PCC is similar to national data. This report supports the continued value of the PCC to both public and acute health care in the state of Kansas.

## Figures and Tables

**Table 1:** Encounter type.

	Number	%
**Exposure**		
Human Exposure	20,713	94.32
Animal Exposure	112	0.51
**Subtotal**	**20,825**	**94.83**
**Non-Exposure Confirmed Cases**		
Human Non-Exposure	87	0.39
**Subtotal**	**87**	**0.39**
**Information Call**		
Drug information	308	1.40
Drug identification	189	0.86
Environmental information	123	0.56
Medical information	30	0.14
Occupational information	1	0.00
Poison information	110	0.50
Prevention / Safety / Education	30	0.14
Teratogenicity information	1	0.00
Other information	49	0.22
Substance Abuse	6	0.03
Administrative	16	0.07
Caller Referred	190	0.86
**Subtotal**	**1,053**	**4.78**
**Total**	**21,965**	**100.00**

**Table 2:** Distribution of human exposures by age and gender.

	Male		Female		Unknown gender		Total		Cumulative Total	
Age (yrs)	N	% of age group total	N	% of age group total	N	% of age group total	N	% of total exposure	N	%
< 1 year	619	52.32	526	47.73	1	0.09	1,183	5.71	1,183	5.71
1 year	1,971	53.26	1,626	46.50	2	0.06	3,701	17.87	4,884	23.58
2 years	1,773	52.39	1,579	46.30	1	0.03	3,384	16.34	8,268	39.92
3 years	852	55.32	681	45.49	3	0.20	1,540	7.43	9,808	47.35
4 years	400	58.48	320	44.02	2	0.28	684	3.30	10,492	50.65
5 years	245	56.71	204	47.11	0	0.00	432	2.09	10,924	52.74
Unknown ≤ 5 years	2	33.33	0	0.00	0	0.00	6	0.03	10,930	52.77
Child 6–12 years	768	61.89	470	39.83	1	0.08	1,241	5.99	12,171	58.76
Teen 13–19 years	620	35.98	990	62.15	2	0.13	1,723	8.32	13,894	67.08
Unknown Child	5	55.56	7	46.67	0	0.00	9	0.04	13,903	67.12
**Subtotal**	**7,255**	**52.18**	**6,403**	**47.58**	**12**	**0.09**	**13,903**	**67.12**	**13,903**	**67.12**
20–29 years	841	47.30	924	52.77	1	0.06	1,778	8.58	15,681	75.71
30–39 years	577	41.72	747	56.12	2	0.15	1,383	6.68	17,064	82.38
40–49 years	447	42.53	558	56.94	3	0.31	1,051	5.07	18,115	87.46
50–59 years	364	40.40	565	57.77	0	0.00	901	4.35	19,016	91.81
60–69 years	292	39.25	411	57.97	1	0.14	744	3.59	19,760	95.40
70–79 years	166	37.22	260	59.50	1	0.23	446	2.15	20,206	97.55
80–89 years	81	33.20	150	64.94	1	0.43	244	1.18	20,450	98.73
≥ 90 years	12	32.43	40	67.80	0	0.00	37	0.18	20,487	98.91
Unknown adult	47	36.43	107	66.88	1	0.63	129	0.62	20,616	99.53
**Subtotal**	**2,827**	**42.11**	**3,762**	**56.69**	**10**	**0.15**	**6,713**	**32.41**	**20,616**	**99.53**
Total*	**10,096**	**48.74**	**10,174**	**50.59**	**26**	**0.13**	**20,713**	**100.00**	**20,713**	**100.00**

* Total includes 97 unknown age cases.

**Table 3:** Origin of call and site exposure for human exposure cases.

Site	Origin of Call		Site of Exposure	
	N	%	N	%
Residence				
Own	14,583	70.41	18,708	90.32
Other	381	1.84	768	3.71
Workplace	324	1.56	395	1.91
Health care facility	4,500	21.73	71	0.34
School	54	0.26	242	1.17
Restaurant / Food service	8	0.04	30	0.14
Public area	63	0.30	181	0.87
Other	775	3.74	164	0.79
Unknown	25	0.12	154	0.74

**Table 4:** Route of human exposures.

	Human exposures		
Route	N	% of All Routes	% of All Cases
Ingestion	17,882	82.44	86.33
Dermal	1,312	6.05	6.33
Inhalation/nasal	1,095	5.05	5.29
Ocular	855	3.94	4.13
Bite/sting	215	0.99	1.04
Unknown	157	0.72	0.76
Parenteral	115	0.53	0.56
Other	25	0.12	0.12
Otic	17	0.08	0.08
Rectal	8	0.04	0.04
Aspiration (with ingestion)	5	0.02	0.02
Vaginal	5	0.02	0.02
**Total Number of Routes**	**21,691**	**100.00**	104.72*

* Some cases may have multiple routes of exposure documented.

**Table 5:** Substance categories most frequently involved in exposures for age ≤ 5 years old.

Substance category	All Substance	%	Single substance exposures	%
Cosmetics/Personal Care Products	1,362	11.89	1,333	12.62
Cleaning Substances (Household)	1,301	11.36	1,259	11.92
Analgesics	1,073	9.37	966	9.14
Foreign Bodies/Toys/Miscellaneous	610	5.32	589	5.57
Antihistamines	590	5.15	537	5.08
Topical Preparations	577	5.04	572	5.41
Vitamins	510	4.45	466	4.41
Dietary Supplements/ Herbals/ Homeopathic	430	3.75	401	3.80
Pesticides	418	3.65	408	3.86
Plants	282	2.46	260	2.46
Gastrointestinal Preparations	276	2.41	246	2.33
Cold and Cough Preparations	250	2.18	228	2.16
Antimicrobials	241	2.10	213	2.02
Hormones and Hormone Antagonists	227	1.98	157	1.49
Cardiovascular Drugs	213	1.86	131	1.24

**Table 6:** Substance categories most frequently involved in exposures of adults (> 20 years old).

Substance category	All substances	%	Single substance exposures	%
Sedative/Hypnotics/Antipsychotics	1,130	11.65	319	6.14
Analgesics	1,103	11.37	508	9.77
Antidepressants	786	8.10	248	4.77
Cardiovascular Drugs	654	6.74	223	4.29
Pesticides	434	4.47	378	7.27
Cleaning Substances (Household)	405	4.18	314	6.04
Alcohols	403	4.15	55	1.06
Anticonvulsants	378	3.90	111	2.14
Antihistamines	333	3.43	151	2.91
Hormones and Hormone Antagonists	272	2.80	135	2.60
Stimulants and Street Drugs	267	2.75	116	2.23
Chemicals	233	2.40	205	3.94
Cosmetics/Personal Care Products	210	2.16	188	3.62
Cold and Cough Preparations	197	2.03	101	1.94
Muscle Relaxants	190	1.96	67	1.29

**Table 7:** Top five most frequent plant exposures.

Botanical Name or Category	N
*Phytolacca americana (L.) (Pokeweed)*	48
Plants: Unknown Toxic Types or Unknown if Toxic	46
*Spathiphyllum species (Peace Lily)*	14
*Philodendron (Species unspecified)*	16
*Cherry (Species unspecified)*	12

**Table 8:** Reason for human exposure cases.

Reason		N	% Human exposures
Unintentional			
	Unintentional - General	11,971	57.8
	Unintentional - Therapeutic error	2,361	11.4
	Unintentional - Misuse	1,226	5.9
	Unintentional - Environmental	625	3.0
	Unintentional - Occupational	238	1.1
	Unintentional - Bite / sting	217	1.0
	Unintentional - Food poisoning	160	0.8
	Unintentional - Unknown	38	0.2
	**Subtotal**	**16,836**	**81.3**
Intentional			
	Intentional - Suspected suicide	2,415	11.7
	Intentional - Misuse	527	2.5
	Intentional - Abuse	348	1.7
	Intentional - Unknown	87	0.4
	**Subtotal**	**3,377**	**16.3**
Adverse Reaction			
	Adverse reaction - Drug	286	1.4
	Adverse reaction - Other	44	0.2
	Adverse reaction - Food	29	0.1
	**Subtotal**	**359**	**1.7**
Unknown			
	Unknown reason	77	0.4
	**Subtotal**	**77**	**0.4**
Other			
	Other - Malicious	43	0.2
	Other - Contamination / Tampering	15	0.1
	Other - Withdrawal	6	0.0
	**Subtotal**	**64**	**0.3**
	**Total**	**20,713**	**100.0**

**Table 9:** Management site of human exposures.

Site of management	N	%
Managed in healthcare facility		
Treated/evaluated and released	3,153	15.2
Admitted to critical care unit	1,281	6.2
Admitted to noncritical care unit	721	3.5
Admitted to psychiatric facility	417	2.0
Patient lost to follow-up / left AMA	175	0.8
**Subtotal (managed in HCF)**	**5,747**	**27.8**
Managed on site, non-health care facility	14,732	71.1
Other	19	0.1
Refused referral	197	1.0
Unknown	18	0.1
**Total**	**20,713**	**100.0**

**Table 10:** Medical outcome of human exposure cases by patient age.

	≤ 5 yrs		6–12 yrs		13–19 yrs		≥ 20 yrs		Unknown child		Unknown adult		Unknown age		Total	
Outcome	N	%	N	%	N	%	N	%	N	%	N	%	N	%	N	%
No effect	4,515	41.31	386	31.10	426	24.72	1,244	18.89	0	0.00	9	6.98	2	2.1	6,582	31.78
Minor effect	1,268	11.60	245	19.74	560	32.50	1,805	27.41	1	11.11	27	20.93	5	5.2	3,911	18.88
Moderate effect	92	0.84	39	3.14	309	17.93	1,112	16.89	0	0.00	2	1.55	69	71.1	1,623	7.84
Major effect	10	0.09	4	0.32	66	3.83	268	4.07	0	0.00	0	0.00	0	0.0	348	1.68
Death	0	0.00	0	0.00	1	0.06	12	0.18	0	0.00	0	0.00	0	0.0	13	0.06
No follow-up, nontoxic	435	3.98	31	2.50	10	0.58	39	0.59	0	0.00	2	1.55	1	1.0	518	2.50
No follow-up, minimal toxicity	4,305	39.39	504	40.61	242	14.05	1,542	23.42	4	44.44	53	41.09	8	8.3	6,658	32.14
No follow-up, potentially toxic	207	1.89	16	1.29	73	4.24	281	4.27	3	33.33	24	18.60	10	10.3	614	2.96
Unrelated effect	98	0.90	16	1.29	36	2.09	279	4.24	1	11.11	12	9.30	2	2.1	444	2.14
Death, indirect report	0	0.00	0	0.00	0	0.00	2	0.03	0	0.00	0	0.00	0	0.0	2	0.01
**Total**	**10,930**	**100.00**	**1,241**	**100.00**	**1,723**	**100.00**	**6,584**	**100.00**	**9**	**100.00**	**129**	**100.00**	**97**	**100.00**	**20,713**	**100.00**

**Table 11:** Medical outcome by reason for exposure in human exposures.

	Unintentional		Intentional		Other		Adverse reaction		Unknown		Total	
Outcome	N	%	N	%	N	%	N	%	N	%	N	%
Death	0	0.00	13	0.38	0	0.00	0	0.00	0	0.00	13	0.06
Death, indirect report	0	0.00	1	0.03	0	0.00	0	0.00	1	1.30	2	0.01
Major effect	53	0.31	273	8.08	0	0.00	9	2.51	13	16.88	348	1.68
Minor effect	2,746	16.31	1,012	29.97	19	29.69	121	33.70	13	16.88	3,911	18.88
Moderate effect	574	3.41	978	28.96	5	7.81	46	12.81	20	25.97	1,623	7.84
No effect	5,836	34.66	720	21.32	7	10.94	14	3.90	5	6.49	6,582	31.78
No follow-up, nontoxic	512	3.04	4	0.12	1	1.56	1	0.28	0	0.00	518	2.50
No follow-up, minimal toxicity	6,399	38.01	146	4.32	17	26.56	92	25.63	4	5.19	6,658	32.14
No follow-up, potentially toxic	391	2.32	189	5.60	7	10.94	16	4.46	11	14.29	614	2.96
Unrelated effect	325	1.93	41	1.21	8	12.50	60	16.71	10	12.99	444	2.14
**Total**	**16,836**	**100.00**	**3,377**	**100.00**	**64**	**100.00**	**359**	**100.00**	**77**	**100.00**	**20,713**	**100.00**

**Table 12a:** Decontamination provided in human exposures by age.

Decontamination	≤ 5 yrs	6–12 yrs	13–19 yrs	≥ 20 yrs	Unknown child	Unknown adult	Unknown age	Total
Cathartic	2	3	40	46	0	0	0	91
Charcoal, multiple doses	1	2	9	5	0	0	0	17
Charcoal, single dose	87	14	176	202	0	0	0	479
Dilute/irrigate/wash	8,317	796	445	2,649	7	58	3	12,275
Food/snack	1,516	142	83	369	0	3	1	2,114
Fresh air	67	35	37	403	3	26	3	574
Lavage	0	0	1	6	0	0	0	7
Other emetic	57	6	4	39	0	1	0	107
Whole bowel irrigation	0	0	1	8	0	0	0	9

**Table 12b:** Therapy provided in human exposures by age.

Therapy	≤ 5 yrs	6–12 yrs	13–19 yrs	≥ 20 yrs	Unknown child	Unknown adult	Unknown age	Total
Alkalinization	4	2	39	143	0	0	0	188
Antiarrhythmic	0	1	0	5	0	0	0	6
Antibiotics	27	10	19	185	0	2	0	243
Anticonvulsants	0	0	2	5	0	0	0	7
Antiemetics	16	9	128	177	0	0	0	330
Antihistamines	19	8	21	86	0	0	1	135
Antihypertensives	0	0	1	18	0	0	0	19
Antivenin (fab fragment)	1	1	2	8	0	0	0	12
Antivenin/antitoxin	0	1	4	10	0	0	0	15
Atropine	0	1	1	12	0	0	0	14
Benzodiazepines	17	7	93	270	0	0	0	387
Bronchodilators	2	5	2	48	0	2	69	128
Calcium	164	8	3	31	0	0	0	206
CPR	0	0	2	7	0	0	0	9
Deferoxamine	0	0	0	2	0	0	0	2
Ethanol	0	0	0	1	0	0	0	1
Extracorp. procedure (other)	0	0	0	1	0	0	0	1
Fab fragments	0	0	0	8	0	0	0	8
Fluids, IV	57	23	490	1,313	0	1	1	1,885
Flumazenil	0	1	6	33	0	0	0	40
Fomepizole	4	0	2	15	0	0	0	21
Glucagon	1	0	4	25	0	0	0	30
Glucose, > 5%	4	0	1	42	0	0	0	47
Hemodialysis	0	0	3	21	0	0	0	24
Hydroxocobalamin	3	1	0	1	0	0	0	5
Hyperbaric oxygen	0	0	0	2	0	0	0	2
Insulin	0	0	1	23	0	0	0	24
Intubation	3	3	27	153	0	0	0	186
Methylene blue	0	0	0	3	0	0	0	3
NAC, IV	1	0	63	105	0	0	0	169
NAC, PO	1	1	14	19	0	0	0	35
Naloxone	5	1	23	131	0	0	0	160
Neuromuscular blocker	2	0	0	6	0	0	0	8
Octreotide	1	0	0	0	0	0	0	1
Other	55	16	99	357	2	3	0	532
Oxygen	9	8	56	379	0	0	69	521
Physostigmine	0	0	4	9	0	0	0	13
Phytonadione	0	0	1	12	0	0	0	13
Sedation (other)	6	5	26	136	0	0	0	173
Sodium thiosulfate	1	0	0	0	0	0	0	1
Steroids	8	2	7	77	0	1	69	164
Vasopressors	0	1	8	65	0	0	0	74
Ventilator	3	3	27	155	0	0	0	188

**Table 13:** Details on deaths and exposure related fatalities.

Age & Gender	Substances	Substance Rank	Cause Rank	Chronicity	Route	Reason
**NON-PHARMACEUTICAL EXPOSURES**						
**Fumes/Gases/Vapors**						
17 year Male	Carbon Monoxide	1	1	Acute	Inhal	Int-S
**Heavy Metals**						
68 year Female	Copper	1	1	Acute	Ingst	Int-S
**PHARMACEUTICAL EXPOSURES**						
**Analgesics**						
73 year Male	Acetaminophen/Hydrocodone	1	1	Acute on Chronic	Ingst	Int-S
	Zolpidem	2	2	Acute on Chronic	Ingst	
**Antihistamines**						
38 year Female	Diphenhydramine	1	1	Acute	Ingst	Int-S
**Cardiovascular Drugs**						
21 year Female	Labetalol	1	1	Unknown	Ingst	Int-S
	Clonazepam	2	2	Unknown	Ingst	
45 year Female	Propranolol	1	1	Acute	Ingst	Int-S
	Valproic Acid	2	2	Acute	Ingst	
	Olanzapine	3	3	Acute	Ingst	
	Bupropion	4	4	Acute	Ingst	
46 year Male	Amlodipine	1	1	Acute on Chronic	Ingst	Int-S
	Lamotrigine	2	2	Acute on Chronic	Ingst	
	Metformin	3	3	Acute on Chronic	Ingst	
	Citalopram	4	4	Acute on Chronic	Ingst	
	Fenobibrate	5	5	Acute on Chronic	Ingst	
	Alpha Blocker	6	6	Acute on Chronic	Ingst	
	Quetiapine	7	7	Acute on Chronic	Ingst	
	Lisinopril	8	8	Acute on Chronic	Ingst	
	Bupropion (Extended Release)	9	9	Acute on Chronic	Ingst	
	Ethanol	10	10	Acute on Chronic	Ingst	
46 year Female	Propranolol	1	1	Acute	Ingst	Int-S
	Trazodone	2	2	Acute	Ingst	
	Paroxetine	3	3	Acute	Ingst	
60 year Male	Carvedilol	1	1	Acute on Chronic	Ingst	Int-S
	Amlodipine	2	2	Acute on Chronic	Ingst	
	Hydrochlorothiazide/ Lisinopril	3	3	Acute on Chronic	Ingst	
	Clopidogrel	4	4	Acute on Chronic	Ingst	
	Duloxetine	5	5	Acute on Chronic	Ingst	
	Acetaminophen/ Hydrocodone	6	6	Acute on Chronic	Ingst	
	Dexlansoprazole	7	7	Acute on Chronic	Ingst	
	Quetiapine	8	8	Acute on Chronic	Ingst	
73 year Female	Metoprolol	1	1	Acute on Chronic	Ingst	Int-S
	Duloxetine	2	2	Acute on Chronic	Ingst	
	Trazodone	3	3	Acute on Chronic	Ingst	
	Donepezil	4	4	Acute on Chronic	Ingst	
	Baclofen	5	5	Acute on Chronic	Ingst	
	Benztropine	6	6	Acute on Chronic	Ingst	
	Lurasidone	7	7	Acute on Chronic	Ingst	
	Alprazolam	8	8	Acute on Chronic	Ingst	
	Zolpidem	9	9	Acute on Chronic	Ingst	
	Meloxicam	10	10	Acute on Chronic	Ingst	
	Salicylate	11	11	Acute on Chronic	Ingst	
	Levothyroxine	12	12	Acute on Chronic	Ingst	
	Omeprazole	13	13	Acute on Chronic	Ingst	
	Vitamin D	14	14	Acute on Chronic	Ingst	
96 year Female	Calcium Antagonist	1	1	Acute	Ingst	Unk
**Cold and Cough Preparations**						
30 year Male	Dextromethorphan/ Guaifenesin	1	1	Acute	Ingst	Int-U
**Electrolytes And Minerals**						
63 year Female	Iron	1	1	Acute on Chronic	Ingst	Int-S
	Ibuprofen	2	2	Acute on Chronic	Ingst	
	Levothyroxine	3	3	Acute on Chronic	Ingst	
**Sedative/Hypnotics/Antipsychotics**						
48 year Female	Quetiapine	1	1	Acute on Chronic	Ingst	Int-S
**Stimulants and Street Drugs**						
20 year Male	Heroin	1	1	Acute on Chronic	Par	Int-A
	Ethanol	2	2	Acute on Chronic	Ingst	

Abbreviations: Inhal: Inhalation; Ingst: Ingestion; Par: Parenteral; Int-S: Intentional-Self; Int-U; Intentional-Unknown; Int-A: Intentional-Another; Unk: Unknown.

**Table 14:** 2015 to 2016 comparison of select statistics.

	2015	2016
**Total Cases**	20,109	21,965
**Calls from Health Care Facility**	4,267	4,514
**Moderate or Major Outcomes**	1,688	1,971
**Deaths**	13	15
